# Respective Prognostic Value of Genomic Grade and Histological Proliferation Markers in Early Stage (pN0) Breast Carcinoma

**DOI:** 10.1371/journal.pone.0035184

**Published:** 2012-04-18

**Authors:** Fabien Reyal, Marc A. Bollet, Martial Caly, David Gentien, Sabrina Carpentier, Hélène Peyro-Saint-Paul, Jean-Yves Pierga, Paul Cottu, Véronique Dieras, Brigitte Sigal-Zafrani, Anne Vincent-Salomon, Xavier Sastre-Garau

**Affiliations:** 1 Département de chirurgie, Institut Curie, Paris, France; 2 Département de radiothérapie, Institut Curie, Paris, France; 3 Département de biologie des tumeurs, Institut Curie, Paris, France; 4 Département de transfert, Institut Curie, Paris, France; 5 Ipsogen SA, Marseille, France; 6 Département d'oncologie médicale, Institut Curie, Paris, France; 7 UMR144, CNRS, Institut Curie, Equipe Oncologie Moléculaire, Paris, France; Health Canada, Canada

## Abstract

**Background:**

Genomic grade (GG) is a 97-gene signature which improves the accuracy and prognostic value of histological grade (HG) in invasive breast carcinoma. Since most of the genes included in the GG are involved in cell proliferation, we performed a retrospective study to compare the prognostic value of GG, Mitotic Index and Ki67 score.

**Methods:**

A series of 163 consecutive breast cancers was retained (pT1–2, pN0, pM0, 10-yr follow-up). GG was computed using MapQuant Dx(R).

**Results:**

GG was low (GG-1) in 48%, high (GG-3) in 31% and equivocal in 21% of cases. For HG-2 tumors, 50% were classified as GG-1, 18% as GG-3 whereas 31% remained equivocal. In a subgroup of 132 ER+/HER2− tumors GG was the most significant prognostic factor in multivariate Cox regression analysis adjusted for age and tumor size (HR = 5.23, p = 0.02).

**Conclusions:**

In a reference comprehensive cancer center setting, compared to histological grade, GG added significant information on cell proliferation in breast cancers. In patients with HG-2 carcinoma, applying the GG to guide the treatment scheme could lead to a reduction in adjuvant therapy prescription. However, based on the results observed and considering (i) the relatively close prognostic values of GG and Ki67, (ii) the reclassification of about 30% of HG-2 tumors as Equivocal GG and (iii) the economical and technical requirements of the MapQuant micro-array GG test, the availability in the near future of a PCR-based Genomic Grade test with improved performances may lead to an introduction in clinical routine of this test for histological grade 2, ER positive, *HER2* negative breast carcinoma.

## Introduction

In early stage breast cancers, biological prognostic factors are essential to provide more accurate information to patients and to guide the indications of adjuvant treatments. Besides Estrogen Receptor (ER), Progesterone Receptor (PR), and *HER2* status, tumor cell proliferation rate is a major biological prognostic factor and predictive factor of response to chemotherapy [1]. However the best assay to assess cell proliferation in clinical practice is still a subject of debate. Mitotic Index (MI) and Ki67 score are widely used. MI, defined as the number of mitoses per 10 high power fields at the periphery of the tumor [2], [3], bears the main part of the prognostic value of the histological grade scoring system in which it is included. This index is linked to both the percentage of tumor cells undergoing mitosis and the duration of the cell-cycle, considering that the M phase is only a short part of the cell-cycle process. However MI does not reflect the doubling time of the tumor. In a large meta-analysis of 85 studies [4], including univariate and multivariate models and involving 7.021 patients, the independent prognostic value of MI for breast cancer patients, regarding the development of metastases or the occurrence of death from cancer as endpoints has been confirmed. Nuclear Ki67 immuno-staining is the other proliferation marker widely assessed in clinical practice. Ki67 protein is present during all active phases of the cell cycle (G1, S, G2, M phases) and is strictly associated with cell proliferation. Ki67 score is most often measured on histological sections and is defined as the percentage of stained invasive carcinoma cells. The prognostic value of Ki67 score has been confirmed in several reviews and meta-analyses including univariate and multivariate models [4], [5], [6], [7]. However its use is hampered by several technical limitations, and no ideal cut-off can so far be recommended for routine use [8].

High throughput molecular analyses are able to provide the expression pattern of genes directly or indirectly linked to tumor cells proliferation. The GG is a 97-gene expression signature designed by Sotiriou *et al* to further discriminate, in the group of histological grade II carcinoma, lesions biologically close to histological grade I carcinoma from those close to grade III [9]. Most of these 97 genes are involved in proliferation and cell cycle control. A high level of concordance between GG and histological grade was observed for histological grade I and histological grade III tumors [9], [10]. In addition, in histological grade II tumors, the GG was able to identify subsets of “HG 1 like” and “HG 3 like” tumors. Consistent with the prognostic value of histological grade, the prognostic value of the GG was confirmed in several studies [9], [11], [12], [13]. Although the GG was primarily designed to improve the discriminatory power of tumor grading of breast cancers, and consistent with the fact that most of the GG genes are linked to proliferation, this genomic index was also reported to be a predictor of relapse in endocrine treated carcinoma [14] and a predictor of histological complete response to primary chemotherapy in ER positive and ER negative breast carcinomas [15]. Since the GG is a genomic measure of cell proliferation, we designed a study to assess the respective prognostic value of the GGI, the MI and the Ki67 score in a series of pN0 early breast cancer patients treated in a large comprehensive cancer centre and followed for at least ten years.

## Materials and Methods

### Patients

The main inclusion criteria for the study were the absence of pathologic axillary lymph node involvement, a follow up above 10 years, and the absence of neoadjuvant therapy before surgery. Using these criteria, 456 early-stage (T1-T2 pN0) breast cancer patients treated between 1995 and 1996 could be retrieved from the Institut Curie database. From these cases, 169 flash-frozen samples stored at −80°C immediately after lumpectomy or mastectomy, and showing more than 50% of tumor cells at the microscopic examination of frozen histological section, were available. The histological features (Histological type, Histological grade assessed according to Elston and Ellis criteria [2], [3], Mitotic Index, Ki67 staining, Estrogen Receptor status, Progesterone Receptor status, *HER2* over expression status) were re-assessed for each sample by pathologists experienced in breast pathology. Tissue samples were fixed in AFA. Tissue sections (4 µm) were prepared from a representative part of each tumour sample to score the following markers: Mitotic Index, Ki67, ER, PR, and HER2.

### Mitotic Index (MI)

Mitotic Index was assessed on histological sections stained by Hematein, Eosin and Saffron. The criteria of Van Diest *et al* were used to define mitotic figures [16], [17]. It corresponds to the mitotic score used in the Nottingham grade; the number of mitoses observed in 10 consecutive high power fields (HPF) using a microscope with a 40× objectives and a 10× ocular was counted. Cut-offs of <10, 10–19 and ≥20 mitosis were used to define low, intermediate and high mitotic indexes.

### Ki67 immunostaining

Tissue sections were first digested in 0.1% trypsin and 0.1% calcium chloride in triphosphate buffer saline pH 7.6 for 5 minutes. Antigen retrieval was performed by incubating tissue sections for 20 minutes in citrate buffer 10 mM (ph 6.1) in a 850 W microwave oven. Tissue sections were then incubated for one hour with the anti-Ki67 monoclonal antibody (Clone MIB1, Dako A/S, Glostrup, Denmark) at 1/100 dilution. The revelation of the staining was performed using the Vectastain Elite ABC peroxydase mouse IgG kit (Vector Burlingame, CA, USA) and diamino-benzidine (Dako A/S) as chromogen. The semiquantitative assessment was performed by estimating at ×200 magnification, the percentage of positive neoplastic nuclei within the area of highest positivity chosen after scanning the entire tumour surface at low power (×10 objective). All nuclei with homogeneous staining even with a light staining or only a nucleolar staining were interpreted as positive. A cut-off of >20% was used to define tumors with high Ki67 score.

### Estrogen Receptor, Progesteron Receptor immunostaining

After rehydration and antigenic retrieval in citrate buffer (10 mM, pH 6.1), the tissue sections were stained for estrogen receptor (ER, clone 6F11, Novocastra, 1/200), and progesterone receptor (PR, clone 1A6, Novocastra, 1/200). Revelation of staining was performed using the Vectastain Elite ABC peroxidase mouse IgG kit (Vector Burlingame, CA) and diaminobenzidine (Dako A/S, Glostrup, Denmark) as chromogen. Positive and negative controls were included in each slide run. Cases were considered positive for ER and PR according to standardized guidelines using a cut-off of ≥10% stained tumour nuclei [18], [19].

### HER2 status

After rehydration and antigenic retrieval in citrate buffer (10 mM, pH 6.1), the tissue sections were stained for HER-2 (clone CB11, Novocastra, 1/1000). Revelation of staining was performed using the Vectastain Elite ABC peroxidase mouse IgG kit (Vector Burlingame, CA) and diaminobenzidine (Dako A/S, Glostrup, Denmark) as chromogen. Positive and negative controls were included in each slide run. The determination of HER2 overexpression was determined according to GEFPICS guidelines with FISH performed in all cases of HER2 2+ result [20].

### Genomic Grade and Genomic Grade Index

All 169 tumour samples available for genomic grade analysis contained more than 50% of cancer cells as assessed by H&E staining on frozen histological section of the samples used for the transcriptome analysis (manufacturer's recommended threshold: 30%). RNA was extracted using Trizol method (Invitrogen) according to manufacturer's instruction and purified using mirRNeasy kit (Qiagen). The concentration, integrity and purity of each RNA sample (260/280, 260/230, 28S/18S, RIN) were measured using RNA 6000 LabChip kit with the Agilent 2100 Bioanalyser (Agilent technologies, Palo Alto, CA). The DNA microarrays used in this study were the Affymetrix HGU133 Plus 2.0 arrays (Affymetrix, Santa Clara, CA). One hundred nanograms of total RNA were used for the IVT express target preparation procotol. Details of the RNA amplification, labeling and hybridization are available from the Affymetrix website (http://www.affymetrix.com). Chips were scanned using the GCS 3000 7G scanner (Affymetrix). Affymetrix quality controls variables were used to check data homogeneity (average noise, average background, percent present, scale factor, degradation slope, GAPDH, β-actin). Outliers defined by 1.5 Inter Quartile Range were flagged for each control. All Genechips had to meet defined QC criteria, and a Genechip was excluded if: 1) an average noise and average background are flagged, 2) or the scale factor and percent present are flagged, 3) or the degradation slope is flagged, 4) or GAPDH and β-actin are flagged. Profiles were normalized using RMAdx procedure. RMA was applied to a reference set of microarrays (191 high-quality profiles), storing the parameters of the RMA fit. To process additional microarrays, these parameters are directly applied, without any re-estimation.

### Genomic Grade Calculation

The Genomic Grade Index (GGI), a continuous variable, was calculated using the MapQuant DxM protocol, based on Sotiriou *et al* [9], and defined as GGI = scale [sum (Probe Sets up in Grade 3 tumors)−sum (Probe Sets up in Grade1 tumors)−offset]. Scale and offset are transformation parameters to standardize the genomic grade index values. The MapQuant GGI was then standardized by setting the scale and offset parameters from a reference dataset (53 ER positive Histological Grade 1 vs 59 ER positive Histological Grade 3), so that the mean GGI of histologic grade 1 tumors was −1 and that of histologic grade 3 tumors was +1. The cut-off was set at 0, with GGI varying between −3 and +3. Based on the value of the GGI, a genomic grade (Genomic Grade 1 or Genomic Grade 3) was then attributed to each tumor sample. To ensure robustness and accuracy of genomic grading, a statistical confidence interval was defined around the cut-off, based on a 3∶1 odds ratio of being GG-1 or GG-3 and validated using precision data. For GGI values into this confidence interval, the genomic grade is defined as ‘equivocal’.

### Statistical Analysis

Baseline characteristics were compared between groups using Chi-square or Fisher's exact tests for categorical variables and Student's t-tests for continuous variables. Overall survival and time to distant metastasis were defined as the time from diagnosis of breast cancer until occurrence of the event. The ten-year follow-up metastastic event predictive performance analysis of histological grade, Genomic Grade Index, Mitotic index and Ki67 was carried out using Area-Under-the-Curve analysis and the ROCR package. Survival analyses were performed using the Kaplan-Meier estimate of the survival function. Comparison between survival curves was performed using the logrank test. Hazard ratios were estimated using the Cox proportional hazard model. P-values were considered significant when below 0.05. Ki67 and GG were analysed first as continuous variables then as categorical variables (the latter corresponding to the routine use of these 2 indexes). Adjustments by patient age and tumor size were used for univariate and multivariate analyses. The analyses were performed using the R software (http://cran.r-project.org).

## Results

### Pathological and Clinical Data

From the 169 cases available for analysis, 163 passed quality controls and constituted the reference cohort. The clinical and pathological features of these 163 cases are summarized in table 1. Tumors corresponded mainly to ductal (78%) or lobular (13.5%) infiltrating carcinoma. All of them were free of axillary lymph node metastases. Tumors were classified as histological grade I in 32.5%, grade II in 43% and grade III in 24.5% of cases. Immunophenotyping showed that ER was expressed in 85.8% (140/163) of the tumors, PR in 68.7% (112/163), HER2 in 6.1% (10/163) whereas 10.4% (17/163) remained negative for the three markers. The median follow-up duration was 154 months (6–182) and 26 patients (17.8%) developed a distant relapse.

**Table 1:** Clinical and Pathological features of 163 pN0 early stage breast carcinomas.

Clinical and Pathological Features. (N = 163)		
Clinical and histological features	Median (min-max)	Number of cases (%)
**Age at Diagnosis (years)**	53 (26–70)	
**Histological Subtype**		
**Infiltrating ductal**		127 (78%)
**Infiltrating lobular**		22 (13.5%)
**Mixed ductal/lobular**		6 (3.7%)
**Others**		8 (5%)
**Pathological Tumor Size (mm)**	20 (7–45)	
**Lympho Vascular invasion**		33 (20%)
**Histological grade**		
**Grade I**		53 (32.5%)
**Grade II**		70 (43%)
**Grade III**		40 (24.5%)
**Number of Mitoses (per ten HPF)**	6 (0–120)	
**Ki67 (percent)**	20 (0–100)	
**ER positive**		140 (85.8%)
**PR positive**		112 (68.7%)
**HER2 positive**		10 (6.1%)
**ER negative PR negative HER2 negative**		17 (10.4%)
**Hormone-therapy**		17 (10.4%)
**Chemotherapy**		11 (6.7%)
**Metastases Events**		29 (17.8%)
**Follow-up (months)**	154 (6–182)	

### Mitotic Index, Ki67 score and Genomic Grade distribution

The Kernel density plot shows a non-continuous and non-homogeneous distribution of Mitotic Index (MI) and of Ki67 score. A majority of samples had a MI below 20 mitoses per ten high power fields and a high MI (≥20) was observed in 21.4% (35/163) of the tumors. A high Ki67 score (>20%) was observed in 43% (70/163) of the tumors (n = 17 with Ki67 = 20%). The GGI showed a continuous and homogeneous distribution (figure S1), ranging from −2.618 to 3.035 with a mean equal to −0.1964 (−2.95 to −1.78, mean = −2.38 for normal breast tissue, data not shown).

### Comparison between the Genomic Grade and the Histological Grade

Genomic Grade (GG) analysis showed that 47.8% of cases were GG-1 (78/163) and 30.7% were GG-3 (50/163). The GG was equivocal in 21.4% of the cases (35/163). The concordance between GG and histological grade was 93% for GG-1 samples (also histological grade I) and 97% for GG-3 (also histological grade III). Among the 70 cases of histological grade II samples, 35 (50%) were reclassified as GG-1 and 13 (18.6%) as GG-3 whereas 31.4% remained equivocal (22/70). Conversely, about two third of the undetermined GG corresponded to histological grade II tumors (22/35). The clinical and pathological features of each subgroup are summarized in tables 2 and 3. The following variables were found significantly correlated to the following combinations of Histological Grade and of Genomic Grade [HG-1_GG-1/HG-2_GG-1/HG-2_GG-3/HG-3_GG-3]: histological type, tumor size, vascular invasion, mitotic index, Ki67 score, ER and PR status, triple negative status, metastatic events. The presence of vascular invasion and Ki67 score were the only factors significantly correlated to the GG when the analysis was restricted to the subgroup of histological grade II tumors.

**Table 2:** Two by two samples distribution.

	GG-1	GG-EQ	GG-3	MI1	MI2	MI3	Ki67 L	Ki67 H
**EE I**	42	8	3	51	2	0	45	8
**EE II**	35	22	13	53	15	2	43	27
**EE III**	1	5	34	0	7	33	5	35
**Ki67 L**	69	19	5	81	8	4		
**Ki67 H**	9	16	45	23	16	31		
**MI1**	72	23	9					
**MI2**	5	9	10					
**MI3**	1	3	31					

	GG-I	GG-EQ	GG-III	HGI	HGII	HGIII
**ER+ HER2−**	73	31	29	52	60	21
**ER−PR−HER2−**	1	0	16	0	3	14
**HER2+**	2	3	5	0	6	4

GG: Genomic Grade. HG: Histological Grade. Ki67 L: Ki67≤20%. Ki67 H: Ki67>20%. MI: Mitotic Index [Elston Ellis. MI1: 0 to 9 mitosis/high power field (hpf). MI2: 10 to 19 mitosis/hpf. MI3>20 mitosis/hpf].

**Table 3:** Clinical and Histological features of pN0 early stage breast carcinomas according to histological grade and to Genomic Grade Index.

Clinical and Pathological features according to Histological Grade and Genomic Grade Index.						
	HG IGG-1	HG IIGG-1	HG IIGG-3	HG IIIGG-3	*p* value	*p* value*
**Number of cases**	42	35	13	34		
**Age (years) Median (Range)**	54 (41–70)	57 (40–70)	55 (40–63)	51 (26–68)	NS	NS
**Histology. N (%)**						
**IDC**	33 (78.5)	24 (68.6)	11 (84.6)	30 (88.2)	5.10^−2^	NS
**ILC**	3 (7)	10 (28.6)	2 (15.4)	2 (5.8)		
**ILC/IDC**	2 (4.7)	1 (2.8)	0	1 (2.9)		
**Others**	4 (9.5)	0	0	1 (2.9)		
**Tumor Size (mm)**	15 (7–35)	20 (10–35)	20 (10–30)	23 (10–45)	2.10^−3^	NS
**Vascular invasion. N (%)**	4 (9)	5 (14.3)	7 (53.8)	8 (23.5)	3.10^−3^	1.4.10^−2^
**High MI (≥20). N (%)**	0	0	1 (7.7)	30 (88.3)	2.10^−16^	NS
**High Ki67 (>20%). N (%)**	3 (7.1)	5 (14.2)	12 (92.3)	30 (88.2)	2.10^−11^	3.10^−4^
**Low MI/low Ki67. N (%)**	39 (92.8)	30 (85.7)	1 (7.6)	0	2.10^−16^	2.10^−4^
**Low MI/high Ki67. N (%)**	3 (7.1)	5 (14.2)	11 (84.6)	4 (11.7)		
**High MI/low Ki67. N (%)**	0	0	0	4 (11.7)		
**High MI/high Ki67. N (%)**	0	0	1 (7.6)	26 (76.4)		
**ER positive. N (%)**	41 (97.6)	33 (94.3)	10 (76.9)	18 (52.9)	6.10^−6^	NS
**PR positive. N (%)**	31 (73.8)	28 (80)	9 (69.2)	12 (35.3)	4.10^−4^	NS
**HER2 positive. N (%)**	0	2 (5.7)	2 (15.3)	3 (8.8)	NS	NS
**ER−/PR−/HER2−. N (%)**	0	1 (2.8)	2 (15.4)	14 (41.2)	5.10^−7^	NS
**Metastatic events. N (%)**	3 (7)	3 (8.5)	3 (23)	9 (26.4)	5.10^−2^	NS

HG: histological grade. GG: Genomic Grade. IDC: Infiltrating Ductal Carcinoma. ILC: Infiltrating Lobular Carcinoma. MI: Mitotic Index. Low MI, MI<20. High MI, MI> = 20. ER: Estrogen Receptor. PR: Progesterone Receptor. Chi-square test, Fisher exact test, Student's t-test *p* values as adapted.

* pvalue in the four groups.

** p value in the group of histological grade II tumors only. HGI GG-EQ, HG-1 GG-3, HG-3 GG-EQ and HG-3 GG-1 samples are excluded from this table.

### Comparison between the Genomic Grade, the Mitotic Index and the Ki67 score

**Figure 1:**
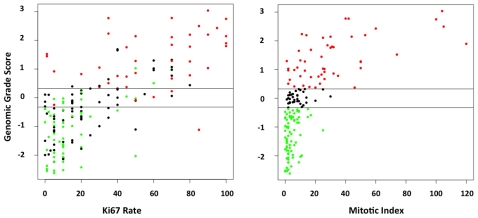
Comparison between Genomic Grade Index, Mitotic Index and Ki67 score. Left. Scatter plot of Genomic Grade Index (GGI) and Ki67 score. Red dot: GG-3 samples; Black dot: GG-Equivocal; Green dot: GG-1. Right. Scatter plot of GGI and Mitotic Index. Red dot: GG-3; Black dot: GG-Eq; Green dot: GG-1. Horizontal stripes: thresholds for GG-1 (upper line) and GG-3 (lower line).

There was a strong positive association between the GG and the MI (Chi-square test, *p* = 2.3.10^−16^). The relation between the continuous value of the GG and the MI is pictured in figure 1. GG-1 samples were classified as MI I in 92.3% (72/78) of the cases and GG-3 samples as MI III in 62% (31/50) cases (table 2). Two thirds (23/35) of the equivocal GG cases corresponded to MI I tumors. GG-3 samples had a low or intermediate MI in 19 out of 50 cases (38%) and only 1 out of 78 (1.3%) GG-1 samples had a high MI.

The Ki67 score was also positively correlated to the GGI. The relationship between the continuous value of the GGI and the Ki67 is pictured in figure 1. It shows a linear interaction pattern of these two variables (R = 0.72, R^2^ = 0.51). GG-1 samples were classified as Ki67 low (≤20%) in 88.4% (69/78) of the cases and GG-3 samples as Ki67 high (>20) in 90% (45/50) (Chi-square test, *p* value =  2.2e-16). Samples with an equivocal GG were classified as Ki67 low in 54.3% (19/35) cases. When the three markers were considered simultaneously, we observed that tumors with high MI (≥20) were almost exclusively classified as GG-3 (30/31, 96.7%) independently of the Ki67 score (table 3). Tumors with low MI (<20) and low Ki67 score (≤20) were almost exclusively classified as GG-1 (69/70, 98.5%). The group of tumors with low MI and high Ki67 score showed a uniform distribution between the 4 subclasses defined by the correlations covariate of Histological and Genomic Grades [HG-1_GG-1/HG-2_GG-1/HG-2_GG-3/HG-3_GG-3]. Interactions between GG, MI, Ki67 score and metastatic events are depicted in figure S2.

### Comparison between the Genomic Grade and immunophenotypic subtypes of breast cancers

A total of 133 samples corresponded to ER positive HER2 negative tumors. Among them, 73 (54.8%) were GG-1, 29 (21.8%) were GG-3 whereas 31 (23.3%) were equivocal GG. In the group of 10 *HER2* positive tumors, two were GG-1, 5 were GG-3 and 3 were undetermined GG. Among the 17 triple negative (ER−, PR−, HER2−) tumors, 1 was classified as GG-1 and 16 as GG-3. It is to be stressed that the proportion of equivocal GGI samples was lower (0%) in the group of triple negative tumors than in HER2 positive (30%) or ER positive (23.3%) subgroups. The status of the histological grade in all of these groups is provided in table 2.

### Prognostic value of proliferation markers

**Figure 2:**
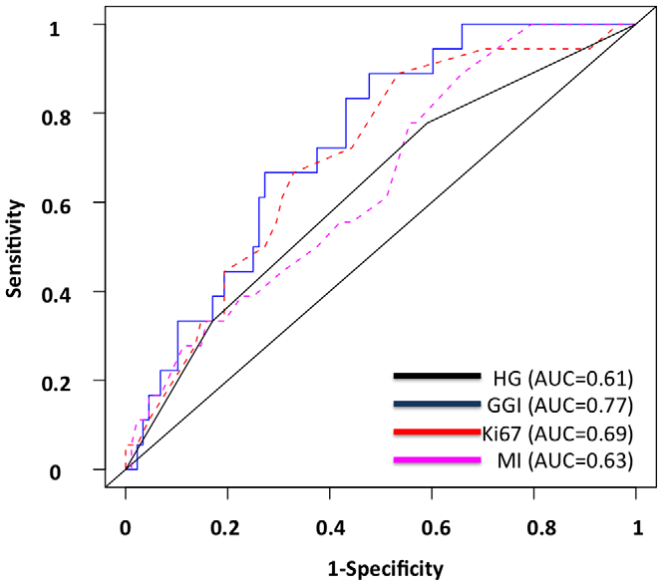
Prediction of metastases events at 10 years of follow-up from initial diagnosis. Receiver Operating Curves. 106 Estrogen Receptor positive HER2 negative samples with 10-year follow-up data. Blue: Genomic Grade Index (GGI); Red: Ki67; Black: Histological Grade (HG); Pink: Mitotic Index (MI).

**Figure 3:**
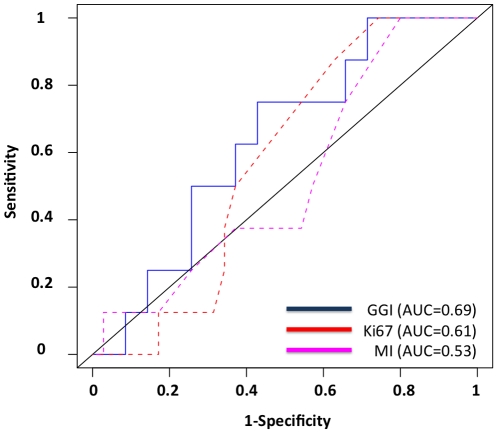
Prediction of metastases events at 10 years of follow-up from initial diagnosis. Receiver Operating Curves. 43 Histological Grade II, ER positive samples with 10-year follow-up data. Blue: Genomic Grade Index; Red: Ki67; Pink: Mitotic Index.

At 10 years of follow-up, the three proliferation markers were found to be associated with the probability of metastatic events. The GGI had the highest Area Under Curves (AUC) values in the overall population of tumors as well as in the different subgroups determined according to histological grade and molecular subtypes immune-phenotype, but there was no significant statistical difference between the three proliferation markers nor with Histologic Grade (Hanley's test) (table 4, figure 2). The largest differences were seen in the group of ER positive *HER2* negative tumors with AUC at 0.77 for GGI [0.66–0.88], 0.69 for Ki67 [0.58–0.80], and 0.63 for MI [0.52–0.74]. In the subgroup of 70 histological grade II tumors, none of the 3 proliferation markers accurately predicted the probability of metastasis (8 events) (table 4, figure 3).

**Table 4:** Prognostic values of Histological Grade, Genomic Grade Index, Ki67 score and Mitotic Index, regarding metastatic events at 10 years of follow-up in biological subgroups of pN0 breast carcinoma.

Prognostic value of HG, GGI, Ki67, MI. AUC (95%CI).					
Tumor groups	N	HG AUC [95%CI]	GGI AUC [95%CI]	Ki67 AUC [95%CI]	MI AUC [95%CI]
**All cases**	106	0.63 [0.57–0.68]	0.74 [0.63–0.85]	0.7 [0.6–0.8]	0.64 [0.54–0.75]
**ER+**	90	0.63 [0.56–0.69]	0.77 [0.67–0.88]	0.71 [0.6–0.81]	0.65 [0.55–0.75]
**ER+/HER2−**	86	0.61 [0.54–0.68]	0.77 [0.66–0.88]	0.69 [0.58–0.8]	0.63 [0.52–0.74]
**HG II**	43	_	0.64 [0.44–0.83]	0.58 [0.45–0.7]	0.52 [0.35–0.7]
**ER+/HG II**	39	_	0.69 [0.49–0.89]	0.61 [0.48–0.75]	0.53 [0.36–0.7]

Receiving Operating Curves. Numbers correspond to Area Under Curves [95% Confidence Interval]. HG: histological grade; GGI: Genomic Grade Index; MI: Mitotic Index; ER: Estrogen Receptor. N: Number.

**Figure 4:**
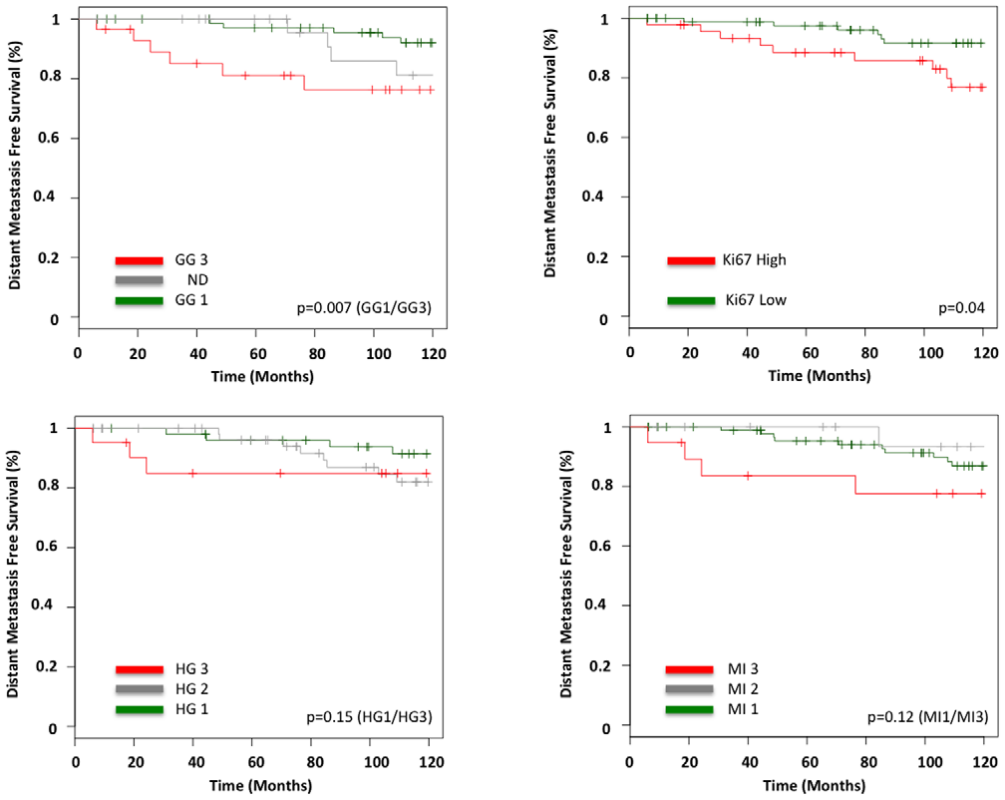
Kaplan Meier Survival Curves. Distant Metastases Free Survival Analysis. 132 Estrogen Receptor positive HER2 negative samples. Logrank tests pvalue. **Top Left.** Genomic Grade (GG). Green: GG-1. Red: GG-3. (*p* = 0.007). **Bottom Left.** Histological Grade (HG). Green: HG-1. Red: HG-3. (*p*  = 0.15). **Top Right.** Ki67 score. Green: Ki67≤20%. Red: Ki67>20%. (*p* = 0.04). **Bottom Right.** Mitotic Index (MI). Green: MI I. Red: MI III. (*p* = 0.12).

Kaplan-Mayer survival curves for 10-year distant metastasis free survival were computed for GG, Ki67 score, MI and histological grade for the ER+/HER2- population (figure 4). GG and Ki67 score were the only significant prognostic factors, with respective relative risks of 4.01 (*p* = 0.007) and 2.56 (*p* = 0.04).

Cox proportional hazard regression analyses using clinical and pathological features with 10-year distant metastasis free survival as endpoint were performed for the 145 cases without systemic chemotherapy and for the subgroup of 126 ER+/HER2-tumors and no systemic chemotherapy. In order to evaluate the biological relation between IHC Ki67 and GG, we performed a multivariate analysis comparing Ki67 score and GGI as continuous variables. It has to be recalled that GG is commercially available as a categorical variable only. The GG was the only significant variable in this model (HR = 2.26; p = 0.014). After adjustment for age at diagnosis and tumor size, in univariate analysis, Ki67 score (continuous and categorical), GG (continuous and categorical), and MI (continuous only) were all significantly correlated to the outcome (table 5). A multivariate analysis was performed to determine if any of the proliferation markers would individually add supplemental prognostic information to that provided by age, tumor size and histological grade. We found that the three markers, GG [categorical: HR = 5.2 (1.3–21) *p* = 0.02, and continuous: HR = 2.36 (1.3–4.2) p = 0.005], Ki67 score [categorical: HR = 3.28 (1.03–10.4) *p* = 0.044] and MI [continuous: HR = 1.07 (1.01–1.14) *p* = 0.024] were the significant prognostic covariates in this model (table 5).

**Table 5:** Prognostic values of Histological Grade, Genomic Grade, Ki67 score and Mitotic Index, regarding metastatic events.

Prognostic values of HG, GG, Ki67 rate and MI								
	HG3 vs HG1		GG3 vs GG1		Ki67 (High vs Low)		MI 3 vs MI 1	
	HR (95%CI)	P	HR (95%CI)	P	HR (95%CI)	P	HR (95%CI)	P
**Univariate**	2.89 (0.6–13.7)	0.18	5.08 (1.5–17.3)	0.009	3.68 (1.3–10.6)	0.017	2.63 (0.7–8.7)	0.11
**Multivariate**	Reference	5.23 (1.3–21)	0.02	3.28 (1.03–10.4)	0.044	7.77 (0.8–70)	NS	

Cox Model, categorical analysis.

HG: histological grade; GG: Genomic Grade; MI: Mitotic Index; ER: Estrogen Receptor. HR: Hazard Ratio. Multivariate Analysis. GG, Ki67 and MI are individually compared to the Histological Grade as defined by Elston Ellis as the gold standard.

### Treatment decision

We analyzed the potential effect of the Genomic Grade on use of adjuvant systemic treatment and hormonal therapy in 70 histological grade II tumors using the current 2010 Institut Curie adjuvant treatment guideline (table 6). When diagnosed in 1995–1996, 11.4% of patients in our cohort actually received hormonal-therapy, 2.8% received hormonal and chemotherapy while 85.7% had no adjuvant treatment. The 2010 treatment decision algorithm was firstly run using the histological grade, and secondly the GG. Equivocal GG samples were considered as histological grade 2. Based on histological grading, 5.7% of patients with histological grade II tumors would have received adjuvant chemotherapy (ACT) alone (all of them were ER−/PR−), 50% would have received adjuvant hormonal therapy (AHT) alone, and 44.3% both treatments. Using the GG, 20% of patients would have been spared of ACT and 15% of AHT. In the ER+/HER2− subgroup (60 patients), 56.6% of patients would have received AHT and 43.3% AHT+ACT based on histological grade; making decision based on GG would have led to a 27% reduction in ACT prescription and to a 16.6% reduction in AHT prescription.

**Table 6:** Adjuvant systemic treatment received by the patients in 1995–1996 and hypothetical treatment decision based on the Institut Curie (IC) 2010 guidelines.

Adjuvant systemic treatment received by the patients in 1995–1996 and hypothetical treatment decision based on the Institut Curie 2010 guidelines.				
Adjuvant Treatment	no AHTno ACT	AHT	AHTACT	ACT
**All HG2 tumors (n = 70)**				
Adjuvant treatment received in 95–96	60 (85.7%)	8 (11.4%)	2 (2.8%)	0 (-)
IC 2010 treatment guidelines with HG	0 (-)	35 (50%)	31 (44.3%)	4 (5.7%)
IC 2010 treatment guidelines with GG	5 (7.1%)	37 (54.8%)	24 (34.3%)	4 (5.7%)
**ER+HER2− HG2 tumors (n = 60)**				
Adjuvant treatment received in 95–96	51 (85%)	8 (13.3%)	1 (1.6%)	0 (-)
IC 2010 treatment guidelines with HG	0 (-)	34 (56.6%)	26 (43.3%)	0 (-)
IC 2010 treatment guidelines with GG	5 (8.3%)	36 (60%)	19 (31.6%)	0 (-)

Treatment decision simulation was based on the histological grade (HG) or the Genomic Grade (GG). AHT: adjuvant hormonal therapy. ACT: adjuvant chemotherapy.

## Discussion

The value of tumor grade as one of the most robust prognostic factors to guide adjuvant treatments in invasive breast cancer, and more specifically in ER+ tumors, is established [21]. The histological grade is used in most of the current treatment decision algorithms [22], [23], [24], [25]. However, about 40–50% of small size node negative breast carcinoma are histological grade II, and there is a lack of definite clinico-biological criteria to guide the indications of adjuvant chemotherapy in this category which currently represents one of the most frequent breast tumors at diagnosis [21]. The Genomic Grade (GG) has been designed to separate histological grade II carcinoma in either low risk (GG-1) or high risk (GG-3) tumors, and therefore address this limitation of histologic tumor grading [9]. At the 2009 St. Gallen consensus conference, the genomic grade has been integrated as a potential adjunct to histological grading and the Ki67 as a prognostic factor. In order to assess the usefulness of the GG in clinical practice compared to that of mitotic index (MI) and of Ki67 score, we have analyzed the respective prognostic value of these parameters in a retrospective study of 163 cases of small size pN0 invasive carcinoma of the breast treated in a single institution between 1995 and 1996. There was a relative correlation between GG, Ki67 score and MI values and these proliferation markers were associated with similar pathological features. A linear correlation was observed between GGI and Ki67. Ki67 gene is one gene out of the 97 genes set that determines the GG signature and they are both measure of proliferation. We also observed a log-linear relation between GGI and MI. Major variations of MI were most commonly observed in tumors with high GGI. Our study confirmed that GG was a significant predictor of distant metastases, more accurately in ER positive breast cancer patients, a result already reported by others [12], [13], [26]. When restricted to the group of histological grade II and ER+/*HER2*− carcinoma (70 samples), GG as well as other proliferation markers did not show any significant prognostic value in term of 10 years metastasis events or in Cox proportional hazard model. This negative result is probably due to the lack of statistical power in this subgroup containing a limited number of cases and events. In a set of 216 cases of histological grade II breast cancers, Sotiriou et al found a better outcome of cases reclassified as GGI I than that reclassified as GGI III [9].

The comparison between the values of proliferation markers and disease outcome showed that GG and Ki67 were of quite comparable prognostic value, higher than that of MI, for 10-year metastasis free survival in this series of small size, node negative, invasive breast carcinoma. In categorical analysis, the value of GG was slightly higher than that of Ki67, but the difference was not statistically significant. The prognostic value of Ki67 in breast cancers has been validated in numerous studies [4], [5], [6], [7]. However, the use of this marker in clinical practice has been hampered by a lack of standardization in technical aspects and in interpretation of the immuno-labeling. For instance, there still is no widely accepted threshold to define high Ki67 score and reported cut-off values vary between 5–30%, more frequently between 10% and 20% [4], [5], [6], [7]. In the present work we used 20% as a threshold for Ki67 score since this is the cut-off set in clinical routine in our institution to discriminate between high and low proliferation tumour. Furthermore, (ii) this value was also the median of Ki67 scores in this series. This threshold has however to be validated on larger series. The inter-observer reproducibility for the scoring of the immuno-labeling has also to be confirmed, especially for values close to the threshold. The use of image analysis systems is likely to overcome this difficulty and to reduce inter-observers variability for equivocal results [27].

In our series, 21% of all tumors, and 31% of Histologic Grade 2 tumors, were classified as Equivocal, i.e. their Genomic Grade Index value fell between the GG-1 or GG-3 categories. The definition of an Equivocal category improves performance of the test around the cut-off point, and enhances the overall robustness of the test, albeit at the expense of the number of samples classified, esp. for Histologic Grade 2 tumors. The proportion of HG-2 cases classified Equivocal could also be driven by the grading performance of each centre, with HG-2 Equivocal cases being higher in reference centres compared to non-expert centres.

By its design and validation, the Genomic Grade represents a genomic measure of tumoral grade, and could therefore be used as direct replacement of the Histologic Grade in current guidelines or treatment algorithms. To explore this option, we replaced HG by GG in the 2010 Institut Curie Treatment Guidelines. We have shown that applying GG to the group of histological grade II and ER+/*HER2*− tumors would have led to a 27% decrease in adjuvant chemotherapy and a 16.6% decrease in hormonal therapy prescriptions. We didn't perform a similar analysis with Ki67 as its position in treatment decision algorithms is not as well as determined as that of tumoral grade. Furthermore Ki67 is not intended to replace tumor grade even if it is closely related to it. The data on Genomic Grade underline the medico-economic impact of the use of gene expression signatures to guide the treatment of breast carcinoma. These figures are consistent with data generated with other gene expression signatures. Using the Recurrence Score (OncotypeDX, Genomic Health), Oratz *et al* showed a switch from adjuvant hormonal therapy to adjuvant chemotherapy in 4% and a switch from adjuvant chemotherapy to adjuvant hormonal therapy in 21% of the cases [28]. For a hypothetical cohort of 100 patients, Hornberger *et al* estimated that a reduction of 200.000$ in treatment costs could be expected using the Recurrence Score [29]. Reference treatment guidelines are however a key driver for the impact of gene expression signatures and prognostic factors in general. Bueno de Mesquita *et al* showed the 70-gene signature would result in a higher use of adjuvant systemic treatment than the rather restrictive Dutch CBO guidelines whereas it would be the opposite when comparing the 70-gene signature to the St. Gallen and Nottingham Prognostic Index guidelines and Adjuvant!Online web-based decision-making tool [30].

Pathological evaluation of breast tumors is based on the use of formalin fixed-paraffin embedded (FFPE) tissues. One limitation to the use of the MapQuant Genomic Grade is the need for a fresh or frozen tissue section. This is not currently routine practice in most breast cancer care centres, while preparing several sections for different assays may represent a challenge with small size tumors (pT1) that represent a growing proportion of cases at diagnosis. Moreover, the DNA micro-array technology on which the MapQuant GG is based requires specific technical platforms and trained teams, and is still currently a high-cost procedure. To address these limitations, an FFPE-based PCR Genomic Grade assay has been developed. A set of 6 genes from the original 97-gene micro-array GG has been selected, based on high reclassification concordance with HG and MapQuant GG. The assay has been validated on a series of 336 breast tumors from a reference breast cancer care centre: 84% of all cases, and 82% of HG-2 tumors, were reclassified as GG-1 or GG-3 (in-house data, and Laios *et al*, abstract SABCS 2011). Additional prognostic validation is ongoing on the BIG-98 study cohort.

In a reference comprehensive cancer centre setting, compared to histological grade, GG added significant information on tumor cell proliferation in breast cancers where adjuvant treatment decision-making remains a challenge. In addition, in patients with histological grade II carcinoma, applying the GG to guide the treatment scheme could lead to a reduction in the prescription of adjuvant hormonal therapy and chemotherapy. However, based on the results observed here and considering (i) the relatively close prognostic values of GG and Ki67, (ii) the reclassification of about 30% of HG-2 tumors as Equivocal GG and (iii) the economical and technical requirements of the MapQuant micro-array GG test, the availability in the near future of a PCR-based Genomic Grade test with improved performances may lead to an introduction in clinical routine of this test for histological grade 2, ER positive, *HER2* negative breast carcinoma

## Supporting Information

Figure S1 **Kernel density plot of the Genomic Grade Index, Mitotic Index and Ki67 score.**
(TIF)

Figure S2 **Comparison between Genomic Grade Index, Mitotic Index and Ki67 score. Correlation to metastatic events. All tumors.** Left: scatter plot of Genomic Grade Index (GGI) and Ki67 score. Red dot: Metastatic progression (29 patients); Black dot: No metastatic progression. Right: scatter plot of GGI and Mitotic Index. Red dot: Metastatic progression (29 patients); Black dot: No metastatic progression.
(TIF)
