# Correction: Respective Prognostic Value of Genomic Grade and Histological Proliferation Markers in Early Stage (pN0) Breast Carcinoma

**DOI:** 10.1371/annotation/46dc7048-61b1-45bd-b4cd-a3b80a2f3f5a

**Published:** 2012-07-10

**Authors:** Fabien Reyal, Marc A. Bollet, Martial Caly, David Gentien, Sabrina Carpentier, Hélène Peyro-Saint-Paul, Jean-Yves Pierga, Paul Cottu, Véronique Dieras, Brigitte Sigal-Zafrani, Anne Vincent-Salomon, Xavier Sastre-Garau

The formatting of Table 5 contained an error. The correct version of Table 5 can be seen here: 

**Figure:**
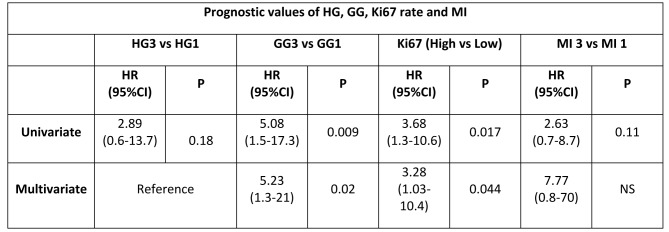


[^] 

