# Supplementary figures and images for: Respective Prognostic Value of Genomic Grade and Histological Proliferation Markers in Early Stage (pN0) Breast Carcinoma
Source: PLoS One. 2012 Apr 18;7(4):e35184. doi: 10.1371/journal.pone.0035184 (PMC3329444; doi:10.1371/journal.pone.0035184)

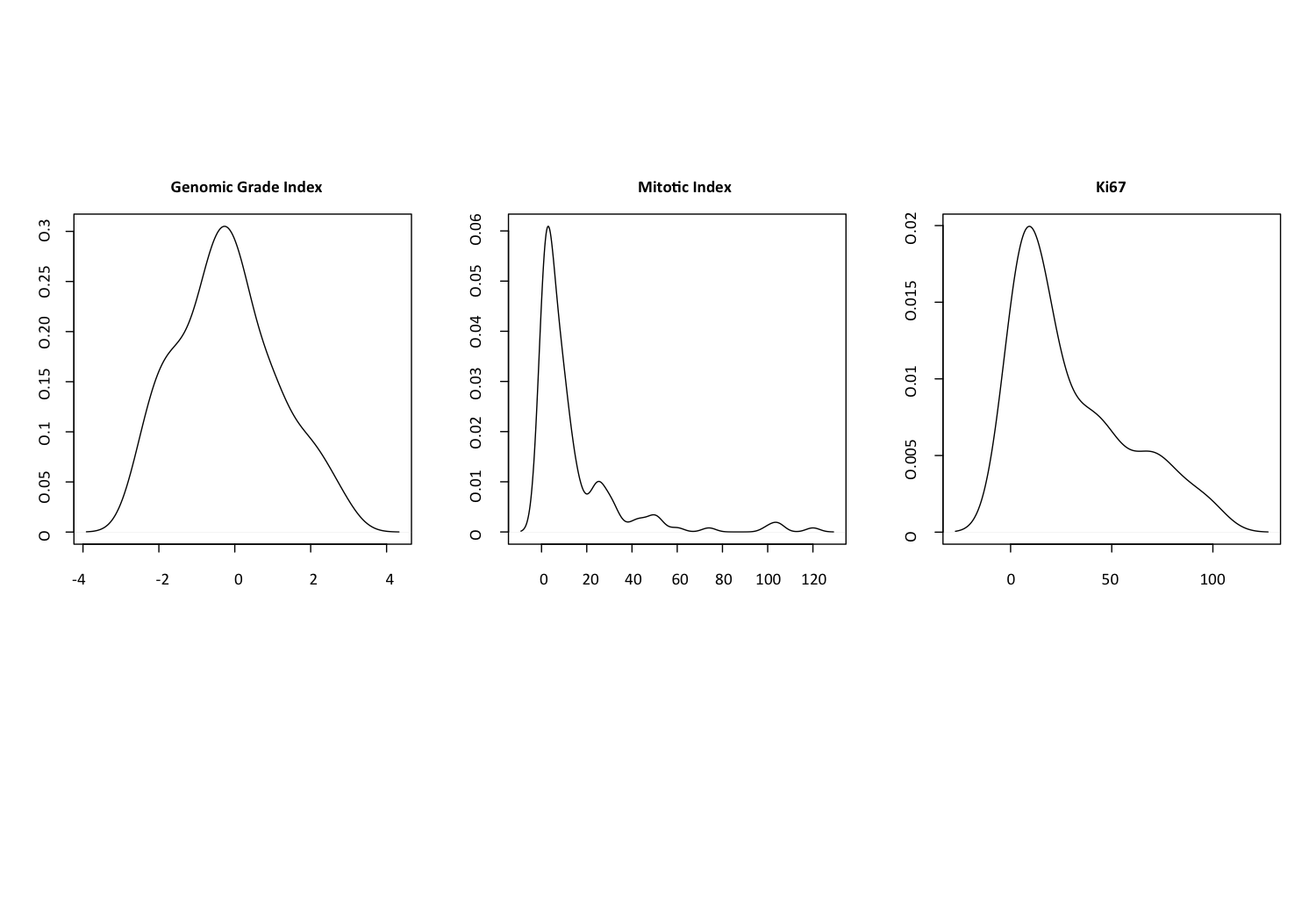

Supplement: Figure S1 — Kernel density plot of the Genomic Grade Index, Mitotic Index and Ki67 score. (TIF) [file pone.0035184.s001.tif]

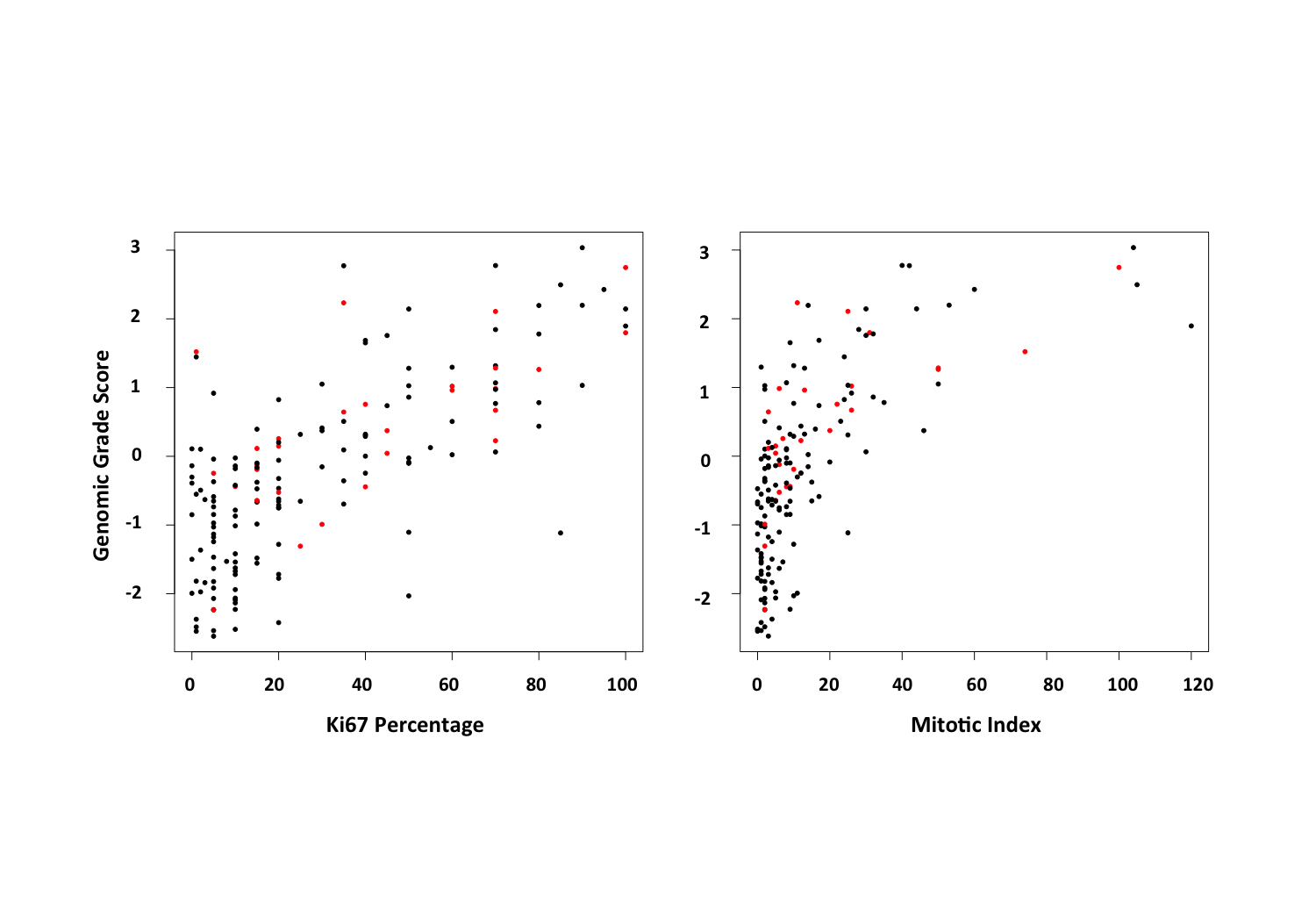

Supplement: Figure S2 — Comparison between Genomic Grade Index, Mitotic Index and Ki67 score. Correlation to metastatic events. All tumors. Left: scatter plot of Genomic Grade Index (GGI) and Ki67 score. Red dot: Metastatic progression (29 patients); Black dot: No metastatic progression. Right: scatter plot of GGI and Mitotic Index. Red dot: Metastatic progression (29 patients); Black dot: No metastatic progression. (TIF) [file pone.0035184.s002.tif]
